# Rate of decline of antibody titers to pandemic influenza A (H1N1-2009) by hemagglutination inhibition and virus microneutralization assays in a cohort of seroconverting adults in Singapore

**DOI:** 10.1186/1471-2334-14-414

**Published:** 2014-07-28

**Authors:** Jung Pu Hsu, Xiahong Zhao, Mark I-Cheng Chen, Alex R Cook, Vernon Lee, Wei Yen Lim, Linda Tan, Ian G Barr, Lili Jiang, Chyi Lin Tan, Meng Chee Phoon, Lin Cui, Raymond Lin, Yee Sin Leo, Vincent T Chow

**Affiliations:** Department of Microbiology, National University Health System, National University of Singapore, Singapore, Singapore; Saw Swee Hock School of Public Health, National University Health System, National University of Singapore, 16 Medical Drive, Kent Ridge, 117597 Singapore, Singapore; Department of Clinical Epidemiology, Tan Tock Seng Hospital, Singapore, Singapore; Department of Infectious Diseases, Tan Tock Seng Hospital, Singapore, Singapore; WHO Collaborating Centre for Reference and Research on Influenza, Melbourne, Australia; National Public Health Laboratory, Ministry of Health, Singapore, Singapore

**Keywords:** Pandemic influenza A(H1N1)pdm09, Declining antibody, Seroconversion, Hemagglutination inhibition assay, Microneutralization assay

## Abstract

**Background:**

The rate of decline of antibody titers to influenza following infection can affect results of serological surveys, and may explain re-infection and recurrent epidemics by the same strain.

**Methods:**

We followed up a cohort who seroconverted on hemagglutination inhibition (HI) antibody titers (≥4-fold increase) to pandemic influenza A(H1N1)pdm09 during a seroincidence study in 2009. Along with the pre-epidemic sample, and the sample from 2009 with the highest HI titer between August and October 2009 (A), two additional blood samples obtained in April 2010 and September 2010 (B and C) were assayed for antibodies to A(H1N1)pdm09 by both HI and virus microneutralization (MN) assays. We analyzed pair-wise mean-fold change in titers and the proportion with HI titers ≥ 40 and MN ≥ 160 (which correlated with a HI titer of 40 in our assays) at the 3 time-points following seroconversion.

**Results:**

A total of 67 participants contributed 3 samples each. From the highest HI titer in 2009 to the last sample in 2010, 2 participants showed increase in titers (by HI and MN), while 63 (94%) and 49 (73%) had reduction in HI and MN titers, respectively. Titers by both assays decreased significantly; while 70.8% and 72.3% of subjects had titers of ≥ 40 and ≥ 160 by HI and MN in 2009, these percentages decreased to 13.9% and 36.9% by September 2010. In 6 participants aged 55 years and older, the decrease was significantly greater than in those aged below 55, so that none of the elderly had HI titers ≥ 40 nor MN titers ≥ 160 by the final sample. Due to this decline in titers, only 23 (35%) of the 65 participants who seroconverted on HI in sample A were found to seroconvert between the pre-epidemic sample and sample C, compared to 53 (90%) of the 59 who seroconverted on MN on Sample A.

**Conclusions:**

We observed marked reduction in titers 1 year after seroconversion by HI, and to a lesser extent by MN. Our findings have implications for re-infections, recurrent epidemics, vaccination strategies, and for cohort studies measuring infection rates by seroconversion.

**Electronic supplementary material:**

The online version of this article (doi:10.1186/1471-2334-14-414) contains supplementary material, which is available to authorized users.

## Background

Recurrent epidemics of influenza occur in winter in temperate climates, whereas in Singapore and other tropical areas, influenza activity is observed all year round with less predictable peaks of activity [1, 2]. Following the emergence of pandemic influenza A(H1N1)pdm09 in March 2009, it was estimated that between 17–31% of participants in a multi-country study were seropositive after the initial epidemic of infections [3]. Despite this, multiple epidemics of infection with the same virus subsequently occurred in Singapore and elsewhere in late 2009 and 2010 [4, 5], with some evidence of shifts in age distribution to involve older individuals [6, 7].

The ability of influenza virus to undergo antigenic drift by its frequent mutations allows it to cause re-infections of the same individual, and also provides one explanation for recurrent epidemics from different strains of the same influenza subtype, thus necessitating frequent updates of influenza vaccines to ensure optimal match between the vaccine and prevailing influenza strains [8]. However, one modeling study suggests that loss of immunity to the same strain also represents an important mechanism underlying the epidemiology of seasonal influenza [9]. One empirical study has shown a decline in antibody titers, with only 52% of naturally infected and 34% of vaccinated individuals having hemagglutination inhibition (HI) titers of ≥ 40 after 6 months of follow-up [10]. However, another smaller study including both naturally infected and vaccinated individuals reported a less dramatic decrease, with only about 5% showing a 4-fold or greater decline in antibody titers after ~1 year by both HI and virus microneutralization (MN) assays [11]. Hence, greater clarity is required on the proportions of individuals still having HI titers of ≥ 40 (which is often considered as the threshold for a seroprotective response) [12, 13], as well as the rate of decline within the period from 6 months to 1 year following seroconversion. Insights are also needed on whether the findings differ when using HI and MN assays. In addition, several cohort studies have suggested that a substantial proportion of influenza infections are asymptomatic [14–17], but it is unclear if the immune responses in such infections are as robust and durable as those observed in symptomatic influenza.

This study aimed to determine the possible waning of post-infection antibodies by quantifying the rate of decline in a cohort of individuals who seroconverted to A(H1N1)pdm09, and were followed up at two additional time-points, i.e. approximately 6 months and 1 year after seroconversion. The study also estimated the proportions with different antibody titers by both HI and MN assays, and explored if there were differences by age, gender and symptoms, as well as how estimates of seroconversion may be affected by longer intervals between serological samples in cohort studies of influenza infection.

## Methods

### Cohort study design and ethical review

Participants in this study were a subset of our previous cohort study, and comprised individuals aged 21 to 75 years from the multi-ethnic cohort of the Singapore Consortium of Cohort Studies [14]. In our previous study, each participant contributed a baseline pre-epidemic sample (obtained between June 2005 to June 2009) and up to two follow-up samples to determine whether they seroconverted to A(H1N1)pdm09 from June to October 2009, when Singapore experienced the initial epidemic of A(H1N1)pdm09 infections. All participants from the original study who consented to continued participation contributed additional blood samples between April and September 2010, as well as answered a questionnaire survey administered concurrently [4]. We then selected the subset of participants who seroconverted (defined as a four-fold or greater increase in titers between any two successive samples by HI assays) to A(H1N1)pdm09 between June and October 2009 to investigate subsequent antibody kinetics in later samples. Participants who reported receipt of influenza vaccine after October 2009 (which would have contained the pandemic A/California/7/2009 strain) were excluded, since this would have altered the subsequent trajectory of antibody titers.

For each participant, we chose the blood sample with the highest HI titer in 2009, which would have been taken (between 20 to 29 August 2009) about 3 weeks after incidence had peaked during the initial epidemic of A(H1N1)pdm09 infections in Singapore, or (between 6 to 11 October 2009) when the epidemic had clearly ended. This was used as a reference point (designated as Sample A), both to detect seroconversion from pre-epidemic samples, as well as to investigate how titers changed over two subsequent samples collected in 2010, as follows:

- Sample B collected from 8 to 22 April 2010, about 6 to 8 months after sample A.
- Sample C collected from 19 to 27 September 2010, approximately 6 months after sample B (and more than 12 to 14 months after sample A).

Finally, we also investigated how any decline in antibody titers might affect the proportion of seroconversions detected had we used longer intervals between sampling time-points, by computing the number with 4-fold or greater rise in titers when independently comparing samples B and C against the pre-epidemic sample taken before the initial epidemic of infections which started in mid-June 2009. The study was approved by the Institutional Review Board of the National University of Singapore.

### Influenza virus and serological assays

All samples (pre-epidemic and samples A to C) were stored at −80°C before analyses by both HI and MN assays. HI assays for the pre-epidemic sample and sample A were performed as a single batch of tests in November 2009 as part of our earlier study on the sero-incidence of A(H1N1)pdm09 during the initial epidemic of infections in Singapore [14]. Samples B and C were batch-tested by HI in December 2010 as part of our follow-up study on subsequent epidemics of A(H1N1)pdm09 in 2010 [4]. The MN assays were conducted in November 2011, as an alternative means of verifying our observations on the marked changes in HI antibody titers found in a preliminary analysis.

For the HI assay, sera from test participants were first pre-treated with receptor-destroying enzyme (Denka Seiken, Tokyo, Japan) at 1:4 (vol/vol) for 16 hours at 37°C, before enzyme inactivation by addition of an equal volume of 1.6% trisodium citrate (Ajax Finechem, Melbourne, Australia) and incubation for 30 minutes at 56°C. The HI assay was then performed according to standard protocols at the World Health Organization Collaborating Center for Reference and Research on Influenza in Melbourne, Australia [18]. Two-fold serial dilutions were first performed on the treated serum in 96-well V-bottom microtiter plates. The pandemic influenza A/California/7/2009 virus was adjusted to 4 hemagglutination units per 25 μl (verified by back titration), and this virus suspension was added to each of the 96 wells before incubating for 30 minutes at room temperature. After this, 25 μl of freshly prepared 1.0% (vol/vol) turkey red blood cells (RBCs) were added at room temperature, and the plates were incubated for 30 minutes for the RBCs to settle. Plates were read and HI titers were obtained from the reciprocal of the highest serum dilution which contained non-agglutinated RBCs. MN assays were performed at the National University of Singapore using pandemic influenza A/Singapore/GP2651/2009 virus isolated in Singapore during the initial epidemic of A(H1N1)pdm09 infections, which has been used in MN work since the latter half of 2009 [19]. Basic Local Alignment Search Tool (BLAST) analyses to compare the sequences of the hemagglutinin and neuraminidase of the two A(H1N1)pdm09 strains (i.e. A/California/7/2009 and A/Singapore/GP2651/2009) revealed nucleotide and amino acid identities of 96-98%, thus confirming their strong homology. Madin-Darby canine kidney (MDCK) cells were seeded with Eagle’s minimum essential medium or EMEM (ATCC, Manassas, VA) in 96-well flat bottom plates and incubated for 24 hours at 37°C with 5% CO_2_ to obtain confluent monolayers. Two-fold serial dilutions of each serum sample were performed using EMEM starting with the 1:10 dilution. Equal volumes of 100 tissue culture infective dose (TCID_50_) of A/Singapore/GP2651/2009 virus (25 μl) and each serum dilution (25 μl) were incubated for 2 hours at 35°C with 5% CO_2_. The cells were washed thrice before adding serum-free medium containing 3 μg/ml of TPCK-treated trypsin (Sigma-Aldrich, St Louis, MO), inoculated with the virus-serum mixtures, and then incubated for 72 hours at 35°C with 5% CO_2_. The neutralizing antibody titer was defined as the reciprocal of the highest serum dilution at which the infectivity of 100 TCID_50_ of virus for MDCK cells was completely neutralized in 50% of the wells.

### Statistical analyses

Geometric mean titers (GMTs) were estimated by assigning a value of 5 for titers below 10, and a value of 1,280 for titers of 1,280 or higher. We then developed a multinomial ordered probit model for the distribution of titers over three sampling periods to allow the rate of change in the distribution of HI and MN titers to be estimated. This formulation permitted age, gender, symptoms as well as interactions with sampling periods, to be adjusted for as potential confounders in the model. Individuals who had fever, sore throat, cough, nasal congestion or shortness of breath were classified as symptomatic infections, while those with none of these symptoms were classified as asymptomatic infections. The likelihood of the data was derived using the following model:

$$x_{ij}\;=\;k+1\cdot \mathrm{if}\times z_{ij}\in \left(\theta_{k},\theta_{k+1}\right)$$

$$z_{ij}\;\sim \;\left(\mu_{ij},\;1\right)$$

$${\mu_{i}}_{j}=\beta^{T}{w_{i}}_{j}+\in_{i}$$

where *x*_*i*j_ is the *j*th observed titer for the *i*th participant, *z*_*i*j_ is a latent variable, a propensity for high titers, and thresholds determining observations, *μ*_*i*j_ controls the location of the latent variable to ensure identifiability, *w*_*i*j_ are individual-level covariates, ∈ _*i*_ is a random effect term, and *θ*_*k*_ are ordered thresholds. Markov chain Monte Carlo sampler was developed to integrate over the space of unobserved latent variables.

Given that our HI assays were performed in two batches and possible intra-laboratory variation of HI titers between batches of replicate assays [20], we conducted a simple sensitivity analysis. We repeated all our statistical analyses based on the assumption that HI titers for samples B and C were up to 2-fold higher than what we measured, and assessed if this would have changed any of our main conclusions.

All statistical analyses were performed using the R statistical software 3.0.0 (Institute for Statistics and Mathematics, Vienna, Austria).

## Results

Of the 838 participants originally enrolled, 727 had at least one additional follow-up blood sample, of which 98 seroconverted to A(H1N1)pdm09. Of the 98 participants, 70 also contributed samples B and C (in April and September 2010). After excluding 3 participants who reported receipt of influenza vaccine between October 2009 and September 2010, we were left with samples from 67 participants for analyses. Participants (30 males and 37 females) had a median age of 42.5 years (range 21 to 62 years), with 53 participants who reported symptoms. Four participants had a 2-fold increase in HI titer, and another 4 different participants had a 2-fold increase in MN titer between successive assays. As a 2-fold change can be considered to be within the margin of error for the respective assays, these observations were retained in subsequent analyses. However, one participant showed a 32-fold increase in HI and 16-fold increase in MN titers between samples A and B, while another showed an 8-fold increase in HI and 16-fold increase in MN titers between samples B and C. Since these two participants may have been re-infected, we excluded them from further analyses.

**Figure 1:**
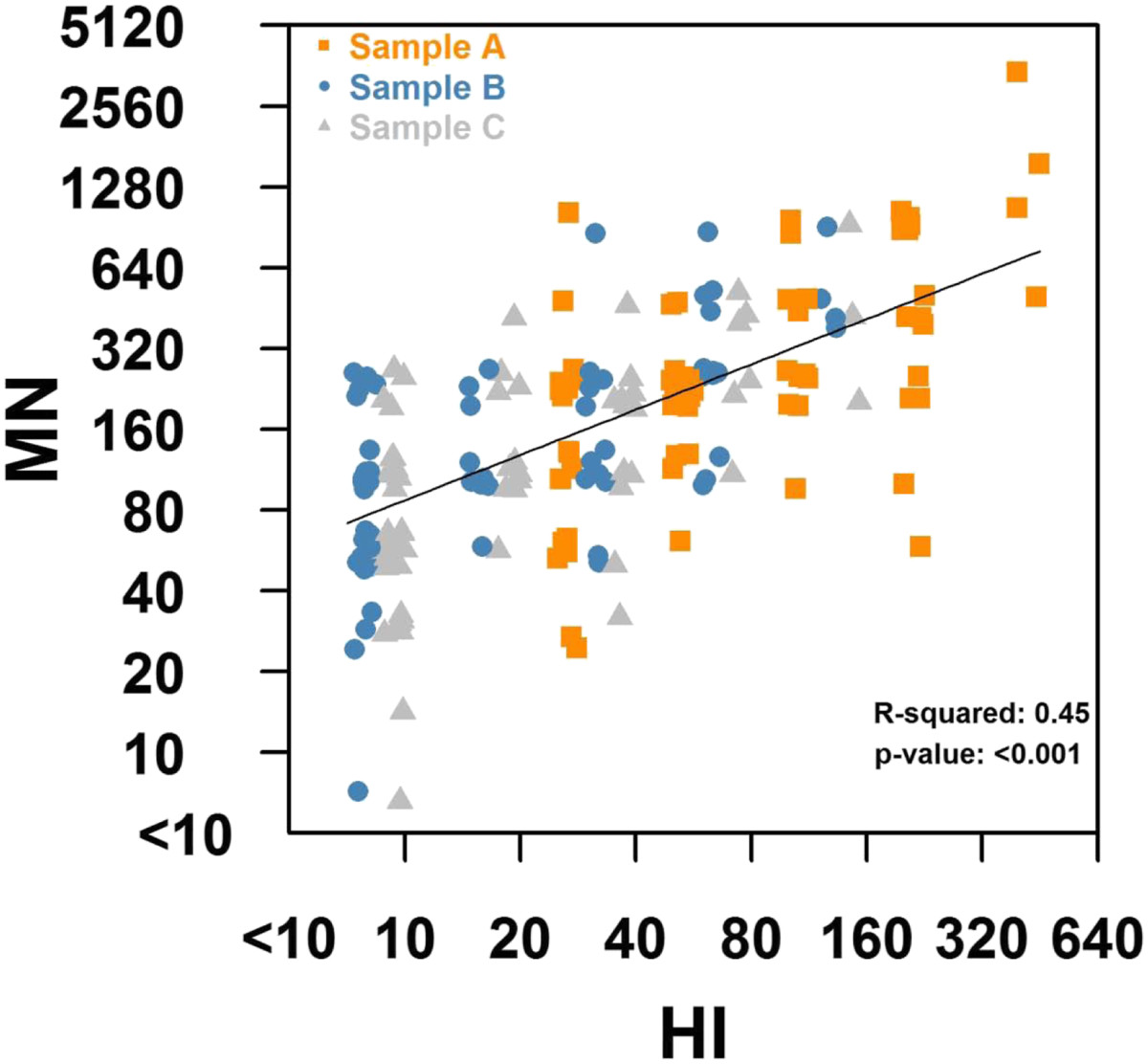
**Scatterplot of titres from HI and MN assays.** Random jitter was added to separate overlapping data points, and different coloured samples denote data from sample **A** (baseline) to sample **C**. The straight line in black is for the best fitting regression model, which has an R-squared of 0.51 (p<0.001).

Figure 1 shows that titers from our HI and MN assays for samples A to C were strongly and significantly correlated (R-squared 0.45, *P* < 0.001), and that the degree of correlation did not vary by sampling period. A HI titer of 40 was approximately equivalent to a MN titer of 160, the former being a commonly referenced correlate of protection in immunological studies. We hence present subsequent MN data with reference to a positive cut-off titer of 160 and above.

**Figure 2:**
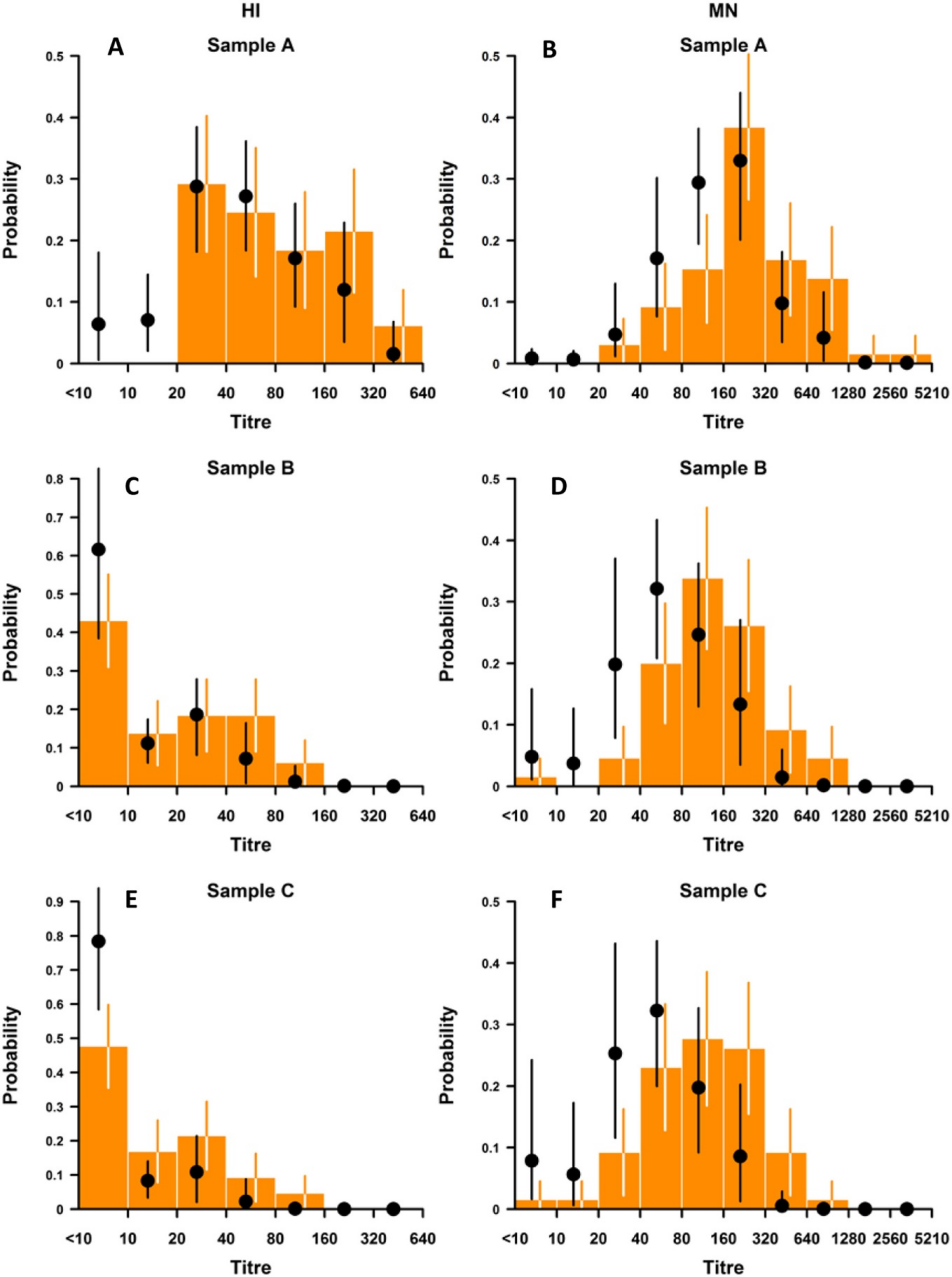
**Model goodness of fit of a multivariate ordered probit model to antibody titre distribution.** In orange are HI assays (left column; Figures 2 **A**, 2 **C** and 2 **E**) and MN assays (right column; Figures 2 **B**, 2 **D** and 2 **F**) at three time points in 2009 (Figures 2 **A** and 2 **B**), April 2010 (Figures 2 **C** and 2 **D**) and September 2010 (Figures 2 **E** and 2 **F**), with 95% confidence interval error bars. In black is the posterior predictive distribution (mean and 95% credible interval). The ordered probit model accounts for age and sex as potential confounders, along with individual random effects and a temporal decay in antibodies, and uses the same θ thresholds at all time points. All the non-Gaussian distribution, and evolving shape of the distribution, are apparent, but the flexibility of the model formulation is able to account for both.

**Figure 3:**
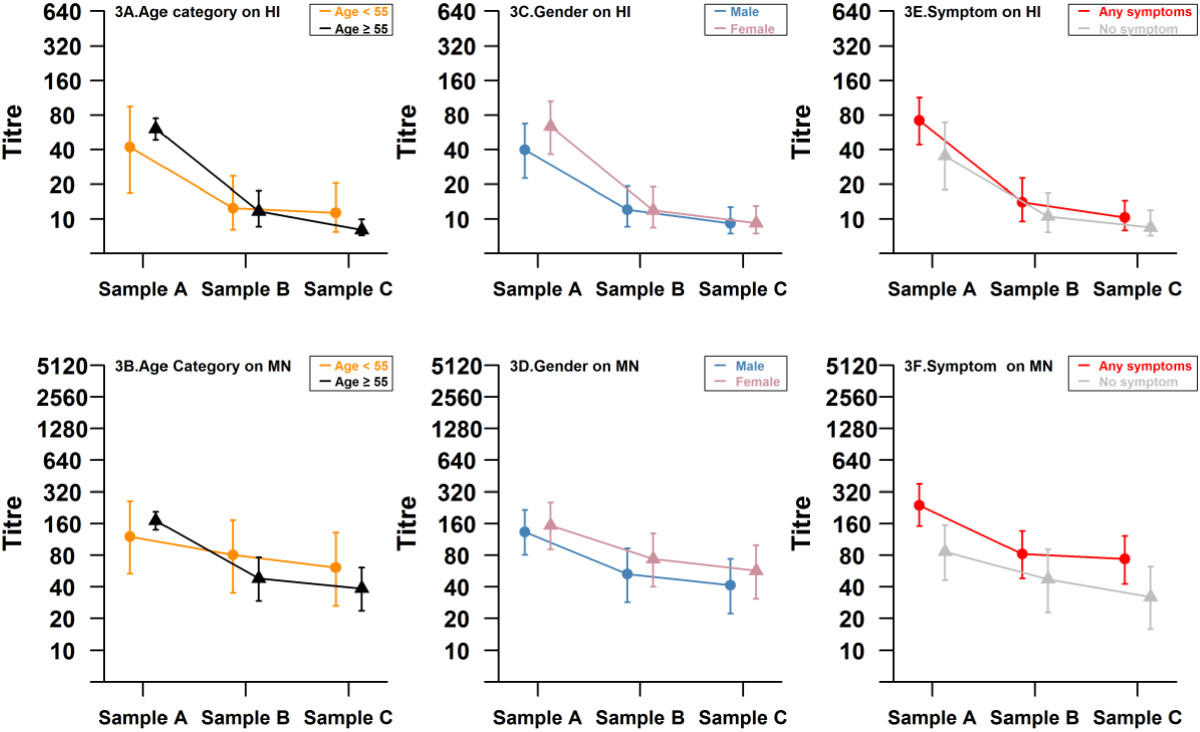
**Modelled antibody trajectories by age category and gender on HI and MN assays.** In orange is the posterior predictive distribution for individuals with age < 55 at three time points in 2009, April 2010 and September 2010, with 95% credible interval error bars (3**A**, 3**B**). In black is the posterior predictive distribution for individuals with age ≥ 55, with 95% credible interval error bars (3**A**, 3**B**). In blue is the posterior predictive distribution for males, with 95% credible interval error bars (3**C**, 3**D**). In pink is the posterior predictive distribution for women, with 95% credible interval error bars (3**C**, 3**D**). In red is the posterior predictive distribution for patients with any respiratory symptoms, with 95% credible interval error bars (3**E**, 3**F**). In grey is the posterior predictive distribution for patients without any respiratory symptoms, with 95% credible interval error bars (3**E**, 3**F**).

Figure 2 presents the observed (bars) and modeled (points) distribution of HI and MN titers from the multinomial ordered probit regression over the 3 sampling periods. It is noted that HI titers in particular are not normally distributed. The chosen model provides a reasonable fit to the data, with predicted 95% credible intervals overlapping with the observed distribution in almost all titer intervals. The model suggests that sampling periods are significantly associated with a negative change in titer by both HI and MN, as indicated by the negative posterior means with 95% credible intervals that are less than zero (Table 1). Female gender (by HI assay) and presence of symptoms (by both HI and MN assays) were also significantly associated with higher titer distributions. There was significant interaction between sampling period and age category, indicating that the older age group (≥55 years) showed a significant decrease in excess of what would be expected for the younger age group for sampling period C by HI assay, and both sampling periods B and C by MN assay. There was also significant interaction between sampling period with gender by the HI assay, and with symptomatic infections by the MN assay. The effect of these interactions is illustrated in Figure 3, which models the mean HI and MN titers with 95% credible intervals. While mean titers were slightly higher in the older age group in sample A by both HI and MN assays (Figures 3A and 3B respectively), the rate of decrease was also faster, such that the trajectory of the mean titers for the two age categories crossed by sample B, and the mean and spread of titers in sample C for those ≥ 55 years was lower than for those aged < 55. We observed that women and symptomatic infections had higher HI titers in sample A, but a marginally faster rate of decline (Figures 3C and 3E), so that titers were not substantially different by gender or symptoms in samples B and C. By MN, the trajectories of antibody decline for the two genders (Figure 3D) were essentially parallel, and while there was a slightly faster rate of decline from samples A to B, symptomatic infections continued to show marginally higher titers in samples B and C due to the substantially higher starting titers in sample A.

**Table 1:** **Results of posterior means with 95% credible intervals of individual-level covariates estimated by the multinomial ordered probit model**

Factor	By HI assay		By MN assay	
	Posterior mean	95% credible interval	Posterior mean	95% credible interval
Age ≥ 55 years (versus age < 55 years)	1.06	(−1.12, 3.53)	1.21	(−1.18, 3.78)
Female (versus male)	1.37	(0.06, 2.71)	0.49	(−0.96, 1.98)
Any symptoms	2.07	(0.38, 3.84)	3.53	(1.60, 5.55)
Sample B (versus sample A)	−2.96	(−4.24, −1.72)	−0.90	(−1.95, 0.18)
Sample C (versus sample A)	−3.54	(−4.96, −2.19)	−2.31	(−3.49, −1.15)
Interaction terms				
Age ≥ 55 years with sample B	−1.28	(−3.02, 0.40)	−2.99	(−4.57, −1.46)
Age ≥ 55 years with sample C	−3.40	(−5.28, −1.57)	−2.77	(−4.30, −1.26)
Female with sample B	−1.44	(−2.37, −0.50)	0.66	(−0.19, 1.50)
Female with sample C	−1.35	(−2.35, −0.37)	0.58	(−0.26, 1.46)
Any symptoms with sample B	−0.66	(−1.89, 0.55)	−1.63	(−2.77, −0.54)
Any symptoms with sample C	−0.53	(−1.84, 0.79)	−0.67	(−1.82, 0.47)

**Figure 4:**
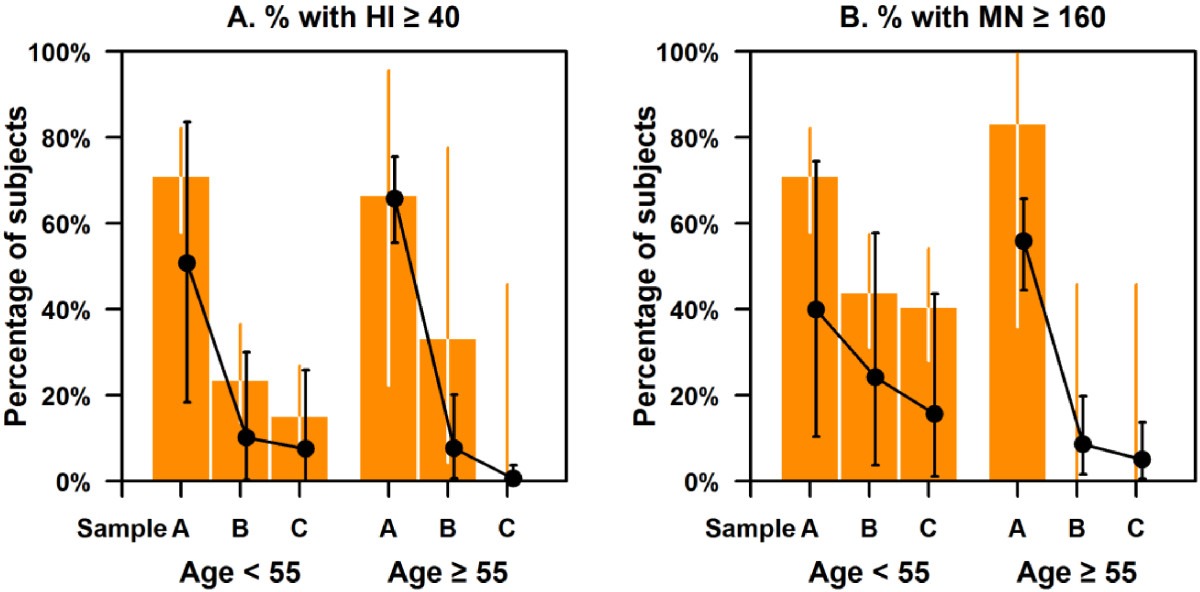
**Estimated and observed proportions by age and sampling period for titres above respective cut-off points for (A) HI ≥40, and (B) MN ≥160.** The orange bars with whiskers, which represent 95% confidence interval, indicate observed proportions stratifying by age and sampling periods. The black points with 95% credible interval error bars represent the estimated proportions adjusting by age and sampling periods.

Figures 4A and B illustrate the fraction which might be below putative cut-off points for seroprotection, both using our model and just the observed proportions. On the observed data, 71% of all subjects had HI titers ≥ 40 and 72% had MN titers ≥ 160 in sample A, but the corresponding proportions were only 25% and 40% by sample B, and 14% and 37% by sample C. None of those aged ≥ 55 years had HI titers ≥40 or MN titers ≥ 160 in sample C, compared with 15% and 41% of those aged < 55 years (*P* = 0.58 and *P* = 0.08 respectively). Results from the modeled distribution were fairly similar, with confidence intervals overlapping those from the observed data.

With HI assays, using longer time intervals between sampling time-points substantially reduced the number of seroconversions detected. Only 27 and 23 participants were found to seroconvert when comparing the titers in samples B and C to the pre-epidemic sample; i.e. only 42% and 35% of the 65 participants who seroconverted by HI with sample A. Of these 65, only 6 did not also seroconvert by MN assays between the pre-epidemic sample and sample A. Of the 59 who seroconverted, 56 (95%) were also assessed to have seroconverted between the pre-epidemic sample and sample B, and 53 (90%) between the pre-epidemic sample and sample C.

In the sensitivity analysis, even assuming that HI titers in samples B and C were up to 2-fold higher than what we measured, we found that there would be significant reduction in titers from samples A to B, and from A to C (Additional file 1: Table S1). There were also no changes to any of the factors and interaction terms identified to be significant in the original regression analysis, and we would still only observe seroconversion in 37 (57%) and 34 (52%) of the 65 individuals respectively when comparing titers of samples B and C versus A (Additional file 1: Table S2).

## Discussion

The objective of our study was to understand temporal changes in antibody titers following seroconversion during the initial epidemic of A(H1N1)pdm09 infections in Singapore. Our results revealed a fairly rapid decline in antibody titers following seroconversion, with only a fifth of those who originally had HI titers of ≥ 40 and half of those with MN titers of ≥ 160 still having titers above the respective cut-off points after a year. There was also some indication that the rate of decline was higher in older individuals, and that the change in antibody titers measured by HI was greater than by MN. Symptomatic infections were associated with higher starting antibody titers, and continued to have marginally higher titers in subsequent samples, at least by MN assays.

Our cohort of participants who seroconverted between June and October 2009, were presumptively infected for the first time with A(H1N1)pdm09 during the initial epidemic in Singapore which peaked in early August 2009 [21]. After its emergence, additional epidemics of A(H1N1)pdm09 were observed in Singapore and elsewhere [4, 7], and mortality records suggests that similar phenomena occurred in past pandemics [22]. Since lower antibody titers do correlate with increased susceptibility [23], the subsequent decline in antibody titers in this cohort offers one possible explanation for re-infections, as well as for recurrent epidemics of A(H1N1)pdm09 in Singapore between November 2009 to February 2010, and April to June 2010 [4]. On the other hand, there have not been large epidemics of A(H1N1)pdm09 in Singapore since then (unpublished data), and the full picture may thus involve a complex interaction of various factors beyond what was observed in our study.

Our findings also have implications for the use of influenza vaccines. Other studies found that vaccination against A(H1N1)pdm09 may induce a weaker response than natural infection [11], and that HI titers decrease significantly in both naturally infected and vaccinated subjects after 6 months [10]. Our previous study on A(H1N1)pdm09 infections confirmed by RT-PCR showed that the proportion with HI titers of ≥ 40 remained reasonably constant in samples collected up to 2 months post-infection, but that a reduction in GMTs was just about to become apparent by then [24]. In combination with findings from this study, it would suggest the most rapid decrease may be occurring between 2 to 6 months after antibody titers peak, with a less marked decline in the window between 6 months to 1 year. If antibody decline in vaccinated subjects indeed follows what we observed in natural infections, then influenza vaccine should be given within 6 months of anticipated exposure. While this is typically the case in temperate countries, the year-round risk of infection in the tropics [2] makes this a much more challenging prospect, and may require more frequent vaccination schedules. In addition, the faster rate of decline in the elderly may exacerbate the above issues with vaccination. Notwithstanding the small number of elderly subjects in our study, the results are consistent with previous understanding on how aging influences the magnitude and quality of the humoral immune response by affecting both the size and diversity of the B-cell repertoire and antibody affinity [25]. A more rapid decline of specific immunity following vaccination may also account for observations on the questionable efficacy of influenza vaccine in older compared to younger individuals [26, 27], as well as shifts in age distribution towards older individuals following a pandemic, which we already observed in later epidemics of A(H1N1)pdm09 infection in Singapore [6] and elsewhere [7]. If indeed we are unable to maintain a durable humoral immune response to influenza in the elderly, then influenza vaccine development would need to focus on alternative strategies such as stimulation of T-cell responses against influenza, as these may be better preserved than humoral immunity in the elderly [28–30]. In addition, our findings also suggest that symptomatic infections may generate more robust immune responses than asymptomatic infections as indicated by the higher titers observed in the former by both HI and MN. However, the data was less clear on whether such higher titers would be sustained over time.

Furthermore, our results also have important implications for the conduct of future sero-epidemiological studies, both using cross-sectional samples and cohort studies. Spacing serum samples more than 6 months apart may substantially underestimate infection rates by seroconversion using HI, depending on the timing of infections relative to the sample collection window, while about 90% of the initial seroconversions by MN could still be detected when using wider sampling intervals. If using a cross-sectional approach to estimate infection rates, we observed that, by samples B and C, only 25% and 14% had HI titers of ≥ 40, which is also used as a possible cut-off for indicating past infection. MN results reflected relatively higher prevalence of neutralizing antibodies, with 40% having titers ≥ 160 after 6 months and 37% after 1 year. More research is warranted to investigate the reasons for the differential rate of decline by HI and MN assays, and whether strain-specific antibody titers by HI or MN better predict protection against infections with the same influenza strain. Some studies comparing MN and HI to measure antibody responses to influenza A(H1N1) have demonstrated greater sensitivity of MN assays in the diagnosis of A(H1N1)pdm09, e.g. 83% versus 71% [11, 13], and this may be related to differential antibody binding characteristics of the two assays.

There were several limitations to our study. Firstly, for logistical reasons, we opted to use different but antigenically homologous A(H1N1)pdm09 strains for our HI and MN assays. Moreover, HI results were based on assays performed in the same laboratory but in two separate batches. However, our sensitivity analysis indicated that our conclusions would remain essentially the same (unless titers in the second batch of HI assays were more than 2-fold higher than what we measured). Notably, while intra-laboratory variation of HI titers of up to 2-fold in replicate assays is not uncommon, variation of more than 2-fold is rare [20]. Another limitation was the relatively small number of older subjects, and we must also acknowledge that our cohort was based on presumptive infections detected by seroconversion on HI assays. It must be remembered that even amongst RT-PCR-positive infections, about 20% do not seroconvert by HI, of which a small proportion do seroconvert by MN [24]. Logistical constraints prevented us from re-testing the entire set of about 2,000 samples from the initial epidemic by MN to identify these subjects. Secondly, we did not conduct follow-up of our subjects to obtain virological samples, and while we excluded 2 participants who were likely re-infected (as indicated by a ≥ 4-fold rise in titer after sample A), the remaining 65 participants could still have included some whose subsequent titers were boosted by repeat exposure or infection. Had we been able to exclude these individuals, we might have found the decrease in titers to be even more dramatic. Finally, we must acknowledge that while our study contributes some knowledge to the rate of decline in antibody titers following initial seroconversion to A(H1N1)pdm09, we are unable to address what might happen following repeated infections with the same strain, repeated infections with different strains of the same subtype, as well as whether the same findings apply to influenza A(H3N2) or influenza B. Our cohort also comprised only healthy adults, and thus we are unable to extrapolate our results to children or individuals with debilitating chronic illnesses.

## Conclusions

Six months and one year after antibodies peaked following presumptive infection with A(H1N1)pdm09, only 25% and 14% of participants respectively had antibody titers against A(H1N1)pdm09 that would be considered protective (HI titer ≥ 40). The decline in antibody titers may explain susceptibility to re-infections, and recurrent epidemics following the initial epidemic of infections during the pandemic. It also suggests that influenza vaccination may have to be administered more frequently in the tropics where there is year-round circulation of influenza viruses. The rate of decline in elderly individuals may be even more rapid, and if our findings are confirmed, may necessitate alternative strategies of influenza vaccine development for this vulnerable group.

## Electronic supplementary material

Additional file 1: Supplementary tables.(DOCX 14 KB)

Below are the links to the authors’ original submitted files for images.

Authors’ original file for figure 1

Authors’ original file for figure 2

Authors’ original file for figure 3

Authors’ original file for figure 4
