# Supplementary material for: Rate of decline of antibody titers to pandemic influenza A (H1N1-2009) by hemagglutination inhibition and virus microneutralization assays in a cohort of seroconverting adults in Singapore
Source: BMC Infect Dis. 2014 Jul 28;14:414. doi: 10.1186/1471-2334-14-414 (PMC4133624; doi:10.1186/1471-2334-14-414)
Supplement: Supplementary file 1 — Additional file 1: Supplementary tables.(DOCX 14 KB) [file 12879_2014_3734_MOESM1_ESM.docx]

**Supplementary tables**

| **Table S1: Original analysis and sensitivity analysis for regression model on HI assay** | | | | |
| --- | --- | --- | --- | --- |
| **Factor** | **Coefficients in original analysis** | | **Coefficients in sensitivity analyses (assumed sample B and C are 2-fold higher)** | |
|  | **Posterior mean** | **95% credible interval** | **Posterior mean** | **95% credible interval** |
| Age ≥55 years (vs age <55 years) | 1.06 | (-1.12, 3.53) | 1.42 | (-0.67, 3.59) |
| Female (vs male) | **1.37** | **(0.06, 2.71)** | **1.26** | **(0.01, 2.56)** |
| Any symptoms | **2.07** | **(0.38, 3.84)** | **2.00** | **(0.46, 3.78)** |
| Sample B (vs sample A) | **-2.96** | **(-4.24, -1.72)** | **-1.10** | **(-2.23, -0.01)** |
| Sample C (vs sample A) | **-3.54** | **(-4.96, -2.19)** | **-1.58** | **(-2.80, -0.45)** |
|  |  |  |  |  |
| Interaction terms |  |  |  |  |
| Age ≥55 years with Sample B | -1.28 | (-3.02, 0.40) | -1.16 | (-2.89, 0.41) |
| Age ≥55 years with Sample C | **-3.40** | **(-5.28, -1.57)** | **-3.19** | **(-5.04, -1.42)** |
| Female with Sample B | **-1.44** | **(-2.37, -0.50)** | **-1.30** | **(-2.22, -0.42)** |
| Female with Sample C | **-1.35** | **(-2.35, -0.37)** | **-1.19** | **(-2.11, -0.27)** |
| Any symptoms with Sample B | -0.66 | (-1.89, 0.55) | -0.60 | (-1.76, 0.55) |
| Any symptoms with Sample C | -0.53 | (-1.84, 0.79) | -0.56 | (-1.73, 0.67) |
| ***Significant results are given in bold font.*** | | | | |

| **Table S2: Original analysis and sensitivity analysis for seroconversion on HI assay** | | | | |
| --- | --- | --- | --- | --- |
|  | **Original analysis** | | **Sensitivity analysis (assume sample B and C are 2-fold higher)** | |
|  | No. seroconverted | % seroconverted | No. seroconverted | % seroconverted |
| **HI assay** |  |  |  |  |
| Sample B vs pre-epidemic | 27 | 41.5% | 37 | 56.9% |
| Sample C vs pre-epidemic | 23 | 35.4% | 34 | 52.3% |
