# Supplementary figures and images for: Rate of decline of antibody titers to pandemic influenza A (H1N1-2009) by hemagglutination inhibition and virus microneutralization assays in a cohort of seroconverting adults in Singapore
Source: BMC Infect Dis. 2014 Jul 28;14:414. doi: 10.1186/1471-2334-14-414 (PMC4133624; doi:10.1186/1471-2334-14-414)

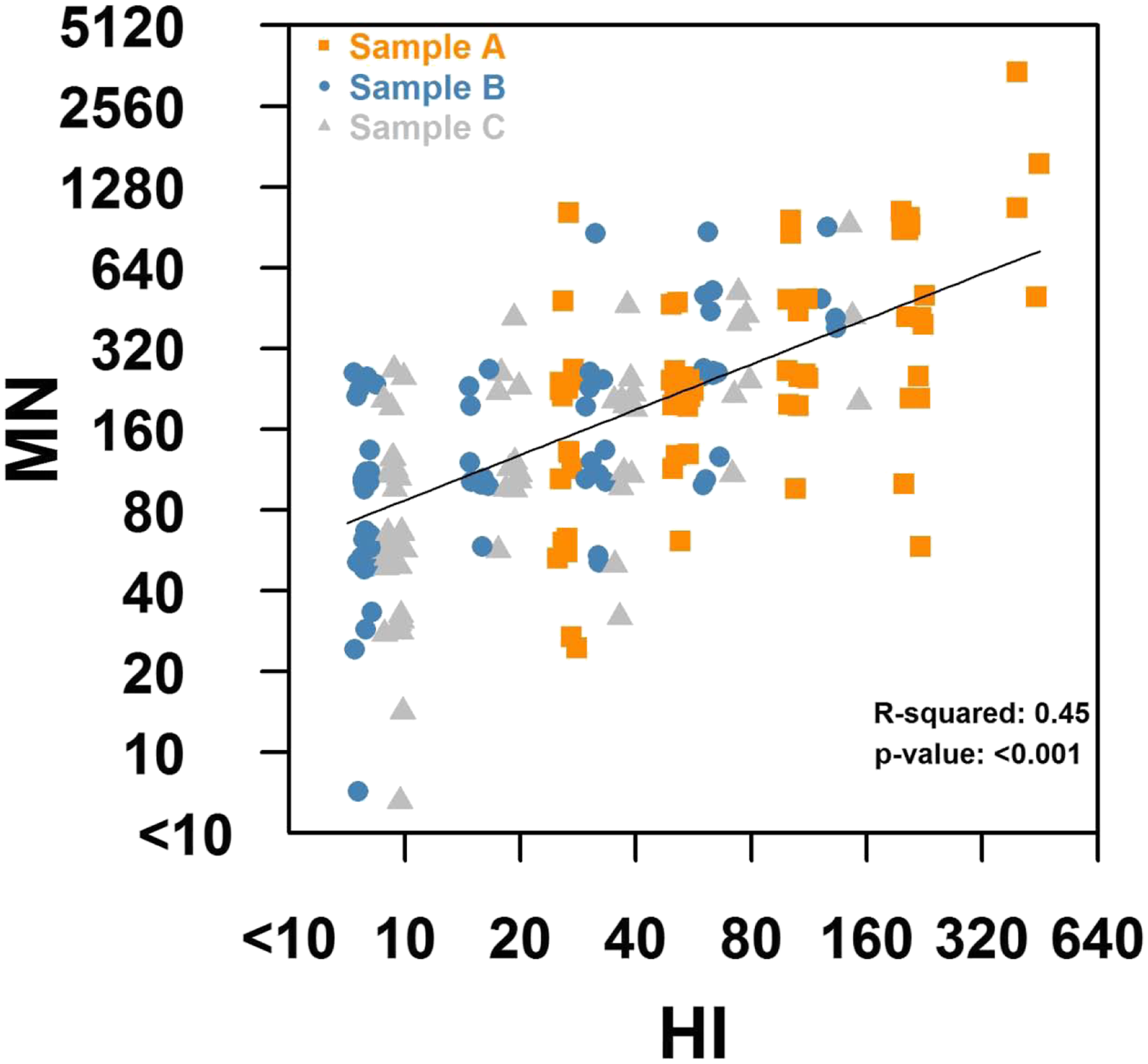

Supplement: Supplementary file 2 — Authors’ original file for figure 1 [file 12879_2014_3734_MOESM2_ESM.tiff]

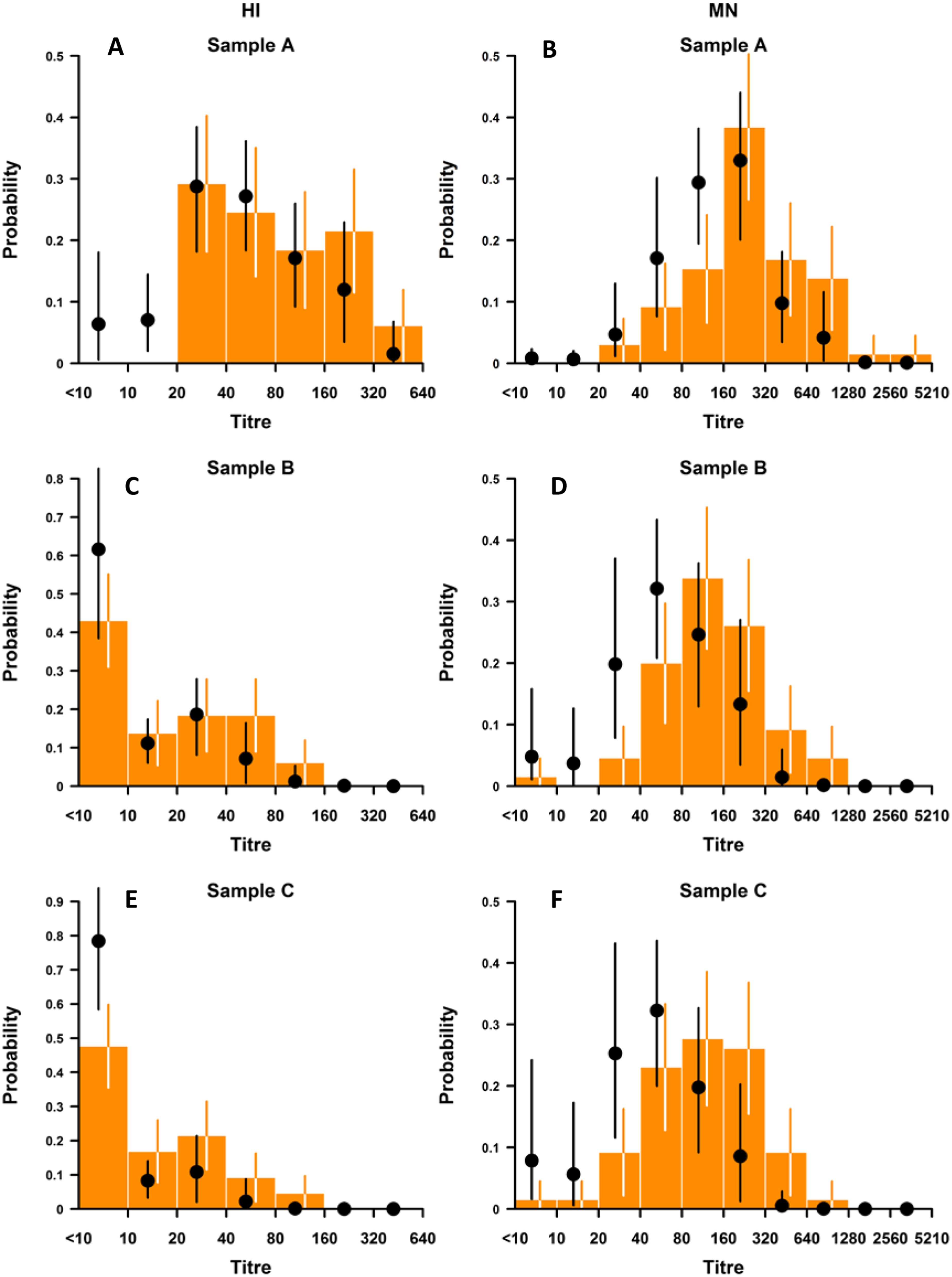

Supplement: Supplementary file 3 — Authors’ original file for figure 2 [file 12879_2014_3734_MOESM3_ESM.tiff]

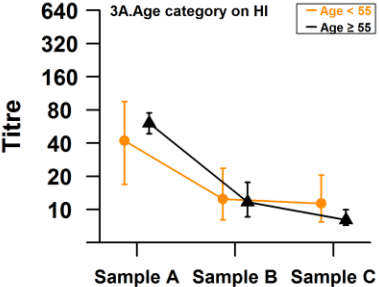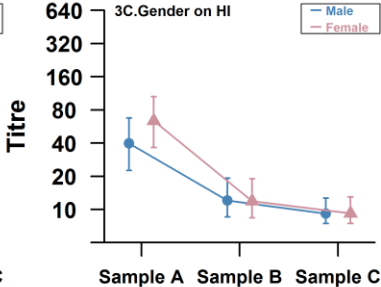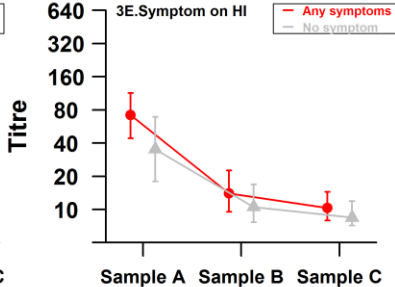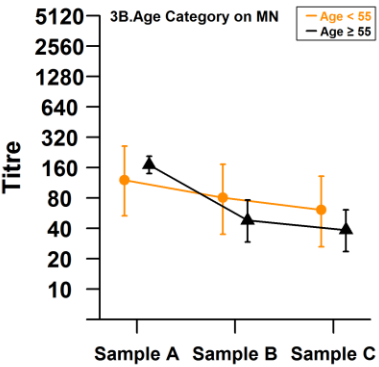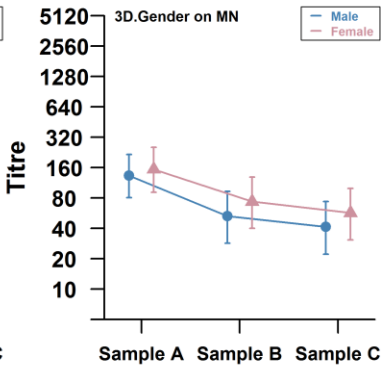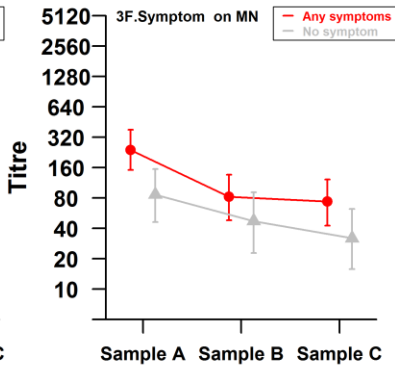

Supplement: Supplementary file 4 — Authors’ original file for figure 3 [file 12879_2014_3734_MOESM4_ESM.pdf]

**A. % with HI  $\geq 40$** 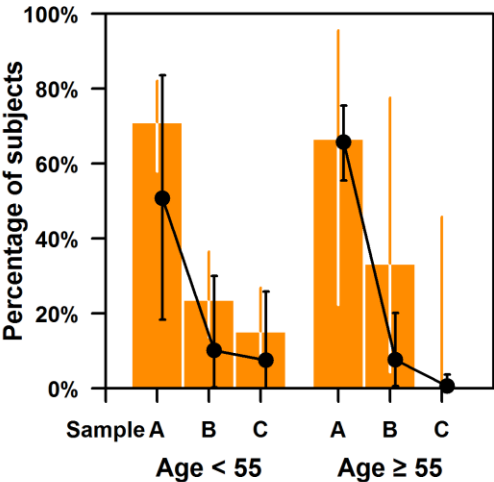**B. % with MN  $\geq 160$** 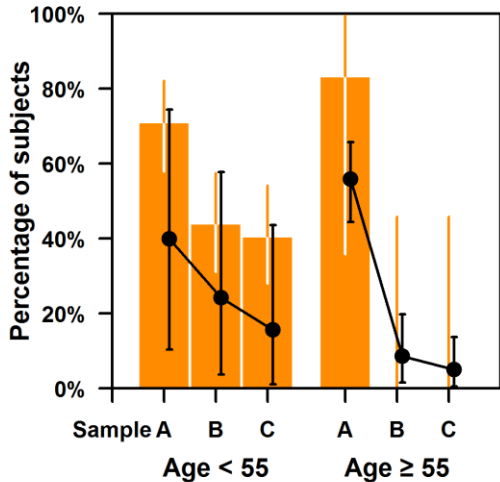

Supplement: Supplementary file 5 — Authors’ original file for figure 4 [file 12879_2014_3734_MOESM5_ESM.pdf]
